# Supplementary material for: An integrated map of fibroblastic populations in human colon mucosa and cancer tissues
Source: Commun Biol. 2022 Dec 3;5:1326. doi: 10.1038/s42003-022-04298-5 (PMC9719516; doi:10.1038/s42003-022-04298-5)
Supplement: Supplementary file 2 — Supplementary Information [file 42003_2022_4298_MOESM2_ESM.pdf]

Supplementary Fig. 1

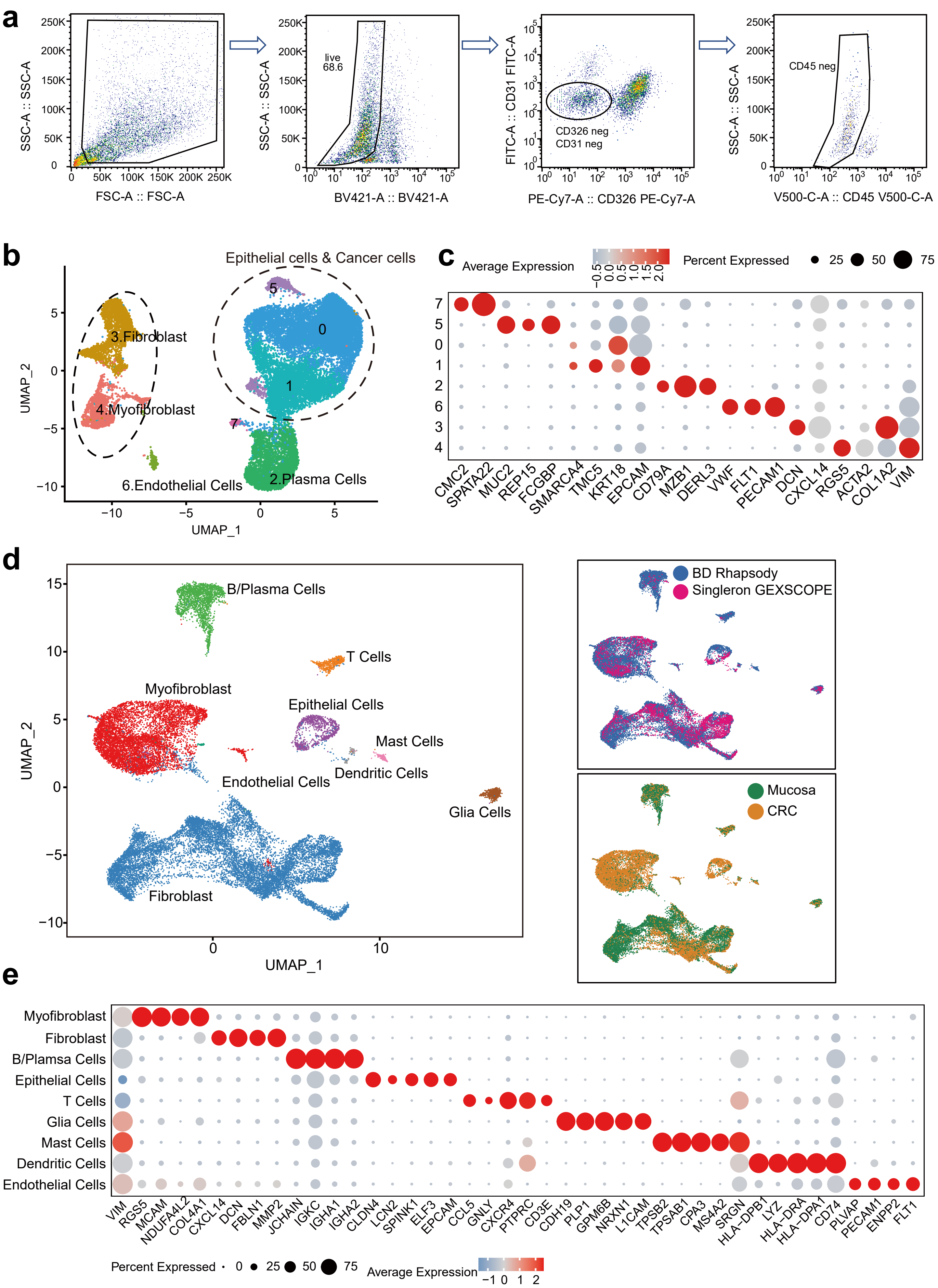

Supplementary Fig. 1: Schematic diagram of cell sorting and data filtering.

a. Flow cytometric sorting of $CD31^-/CD326^-/CD45^-$ fibroblasts. **b.** UMAP projection of $CD45^-$ sorted normal mucosa and colon cancer cells which from patient 10,11,12 and were constructed libraries with Singleron GEXSCOPE. **c.** Dot plots shows the expression of markers of each cluster in Supplementary Fig. 1b. **d.** Pre-analysis of all sorted stromal cells. Identity of cell types have been annotated on the UMAP projection (left). Cells from different experiments (right, top) and sources (right, bottom) are labeled by different color on the UMAP projection. **e.** Dot plots shows the expression of markers of each cluster in Supplementary Fig. 1d.

Supplementary Fig. 2

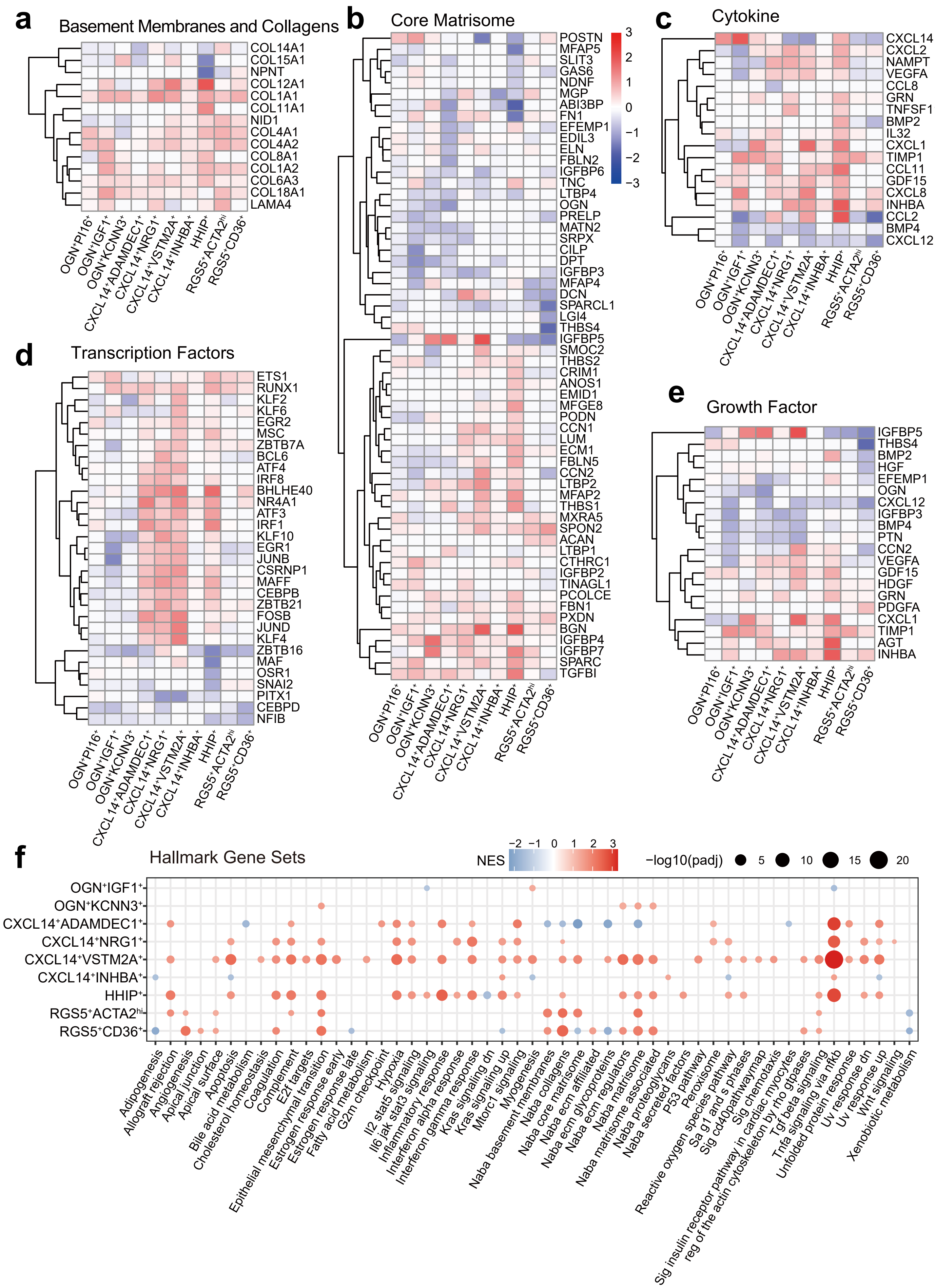

Supplementary Fig. 2: Differential expression in fibroblasts and myofibroblasts between human colonic mucosa and human colon cancer tissues.

a, b, c, d, e. Heatmap showing the differentially expressed genes (DEGs) between cancer fibroblastic cells and colonic mucosa fibroblastic cells for each cluster. The gene sets used are the same as in Fig. 3. The gradient color bar indicates avg_log2FC of colon cancer tissues relative to colonic mucosa. The selected gene sets: **a.** Genes encoding structural components of basement membranes (M5887) and collagen proteins (M3005). **b.** Genes encoding core extracellular matrix components including ECM glycoproteins, collagens and proteoglycans (M5884). **c**. Genes encoding cytokines (GO:0005125). **d**. Genes encoding growth factors (GO:0008083). **e.** Genes encoding transcription factors (http://humantfs.ccbr.utoronto.ca). **f.** Gene set enrichment analysis (GSEA) analysis of Hallmark Gene Sets of clusters DEGs between CRC and mucosa. The color bar represents the normalized enrichment score (NES).

Supplementary Fig. 3

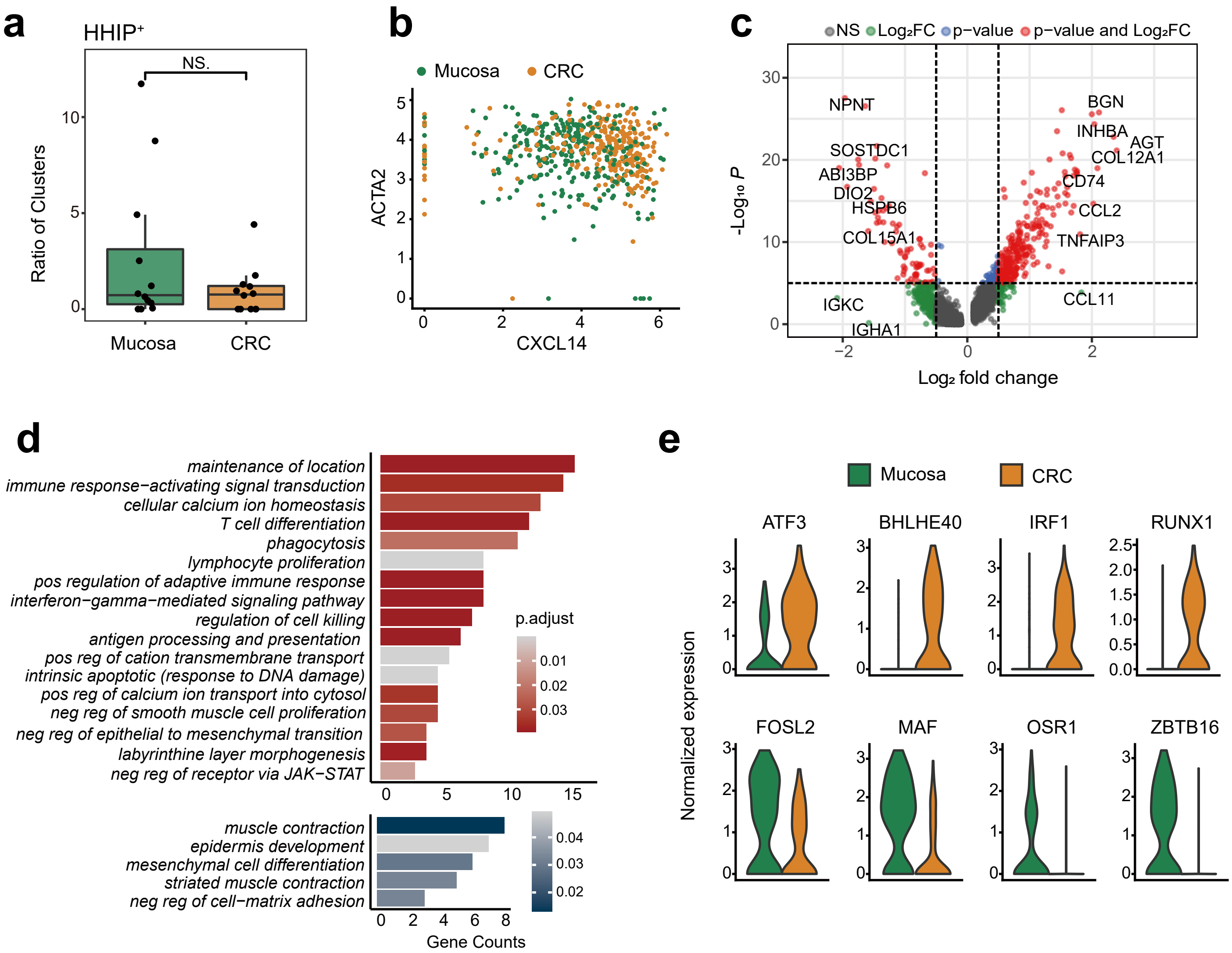

Supplementary Fig. 3: Differential expression of the HHIP⁺ cluster in human colon mucosa and in colon cancer tissues.

a. Boxplot showing the proportion of HHIP⁺ fibroblasts in mucosa and colon cancer tissues. The p value was calculated by the t test. **b.** Scatter plot showing the cells in HHIP+ cluster were ACTA2 and CXCL14 double positive in mucosa and CRC. Each point represents a cell, and the x and y axes represent the normalized expression *CXCL14* and *ACTA2*, respectively. **c.** Volcano plot showing differentially expressed genes in the HHIP⁺ cluster between mucosa and colon cancer tissues. The most significantly different genes are labeled in the plot. **d.** Gene Ontology analysis of differentially expressed genes for the HHIP⁺ cluster. The red bar (top) shows the enrichment of genes elevated in colon cancer tissues; conversely, the blue bar (bottom) shows the enrichment of genes with lower expression in colon cancer tissues. **e.** Violin plot showing the expression of several significantly differentially expressed transcription factors in HHIP⁺ fibroblasts in mucosa and colon cancer tissues.

Supplementary Fig. 4

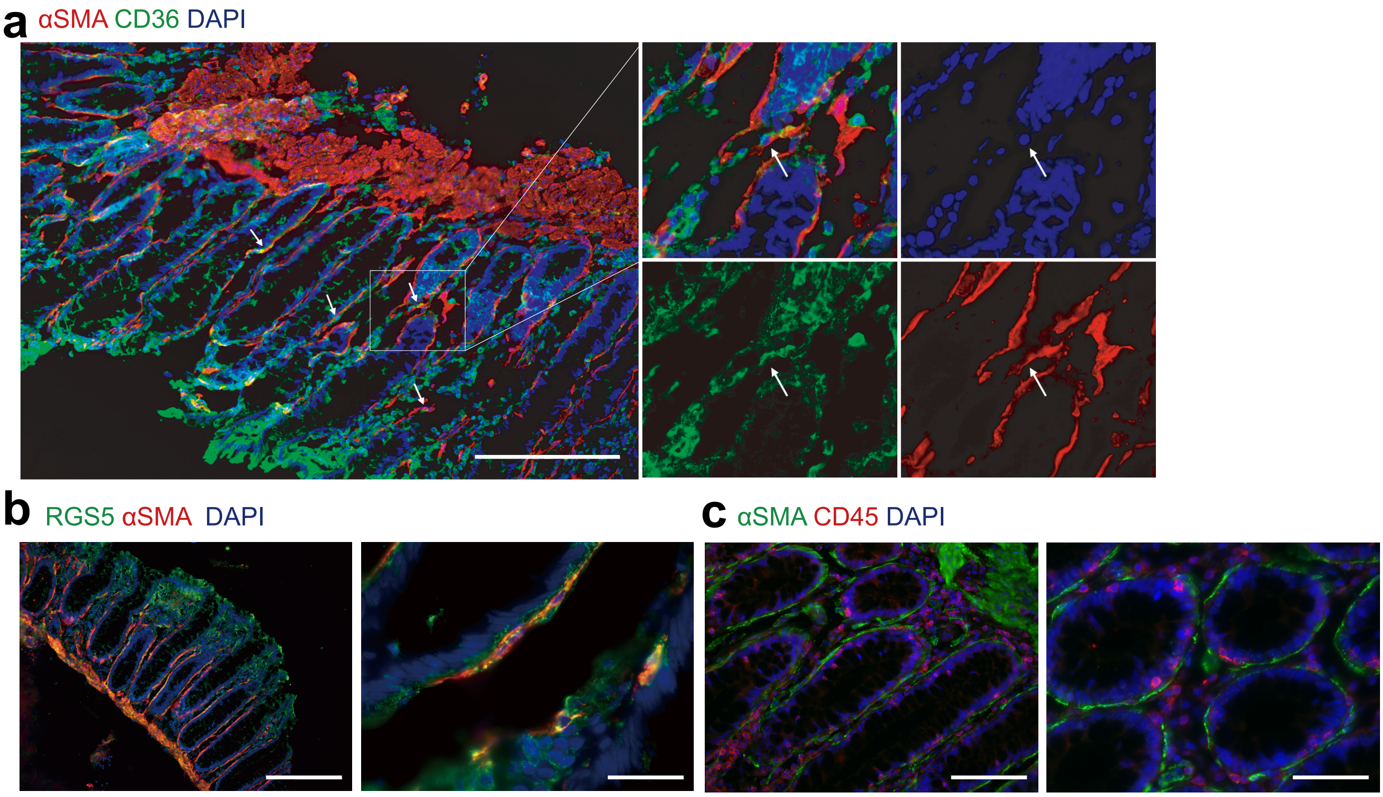

Supplementary Fig. 4: Immunofluorescence staining of myofibroblasts in human colon mucosa. **a.** ACTA2⁺CD36⁺ fibroblasts in normal mucosa labeled by a-SMA (red) and CD36 (green). Scale bar indicates 200 mm. RGS5, Regulator of G Protein Signaling 5, encoded by RGS5 gene. CD36 Molecule, encoded by CD36 gene. **b.** Double labeling for a-SMA (red) and RGS5 (green). left: vertical section, scale bar indicates 500 mm; right: detailed view, scale bar indicates 20 mm. **c.** Double labeling for a-SMA (green) and CD45 (red). left: vertical section, scale bar indicates 100 mm; right: cross section, scale bar indicates 50 mm.

Supplementary Fig. 5

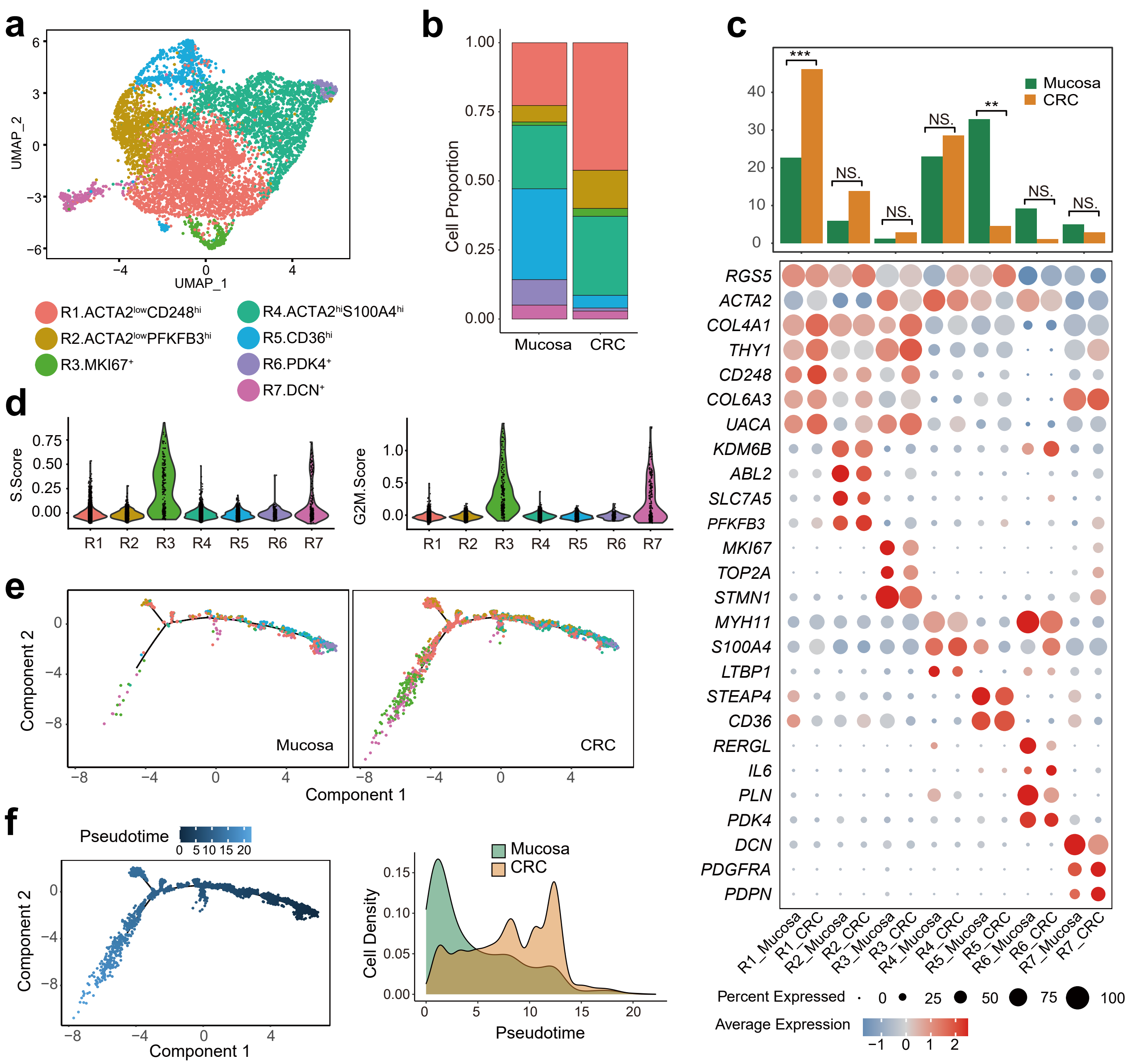

Supplementary Fig. 5: RGS5⁺ fibroblast proportions increased in human colon cancer tissues. **a.** UMAP projection of further analysis of RGS5⁺ clusters. Cells were colored by new cluster identity. **b.** Comparison of RGS5⁺ fibroblast composition between human colon mucosa and colon cancer tissues. **c.** Dot plot showing the scaled expression of signature genes for each RGS5⁺ subcluster in human colon mucosa and colon cancer tissues. The top histogram shows the proportional change in each subcluster. The p value was calculated by the t test. **d**. Violin plots showing the G2M.Score and S. Score of each subcluster. **e.** Trajectories of RGS5⁺ fibroblasts, displayed separately by sample source and colored by subcluster identity. **f.** left: Trajectories of RGS5⁺ fibroblasts color with pseudotime. right: Density of mucosal and colon cancer RGS5⁺ fibroblasts along the pseudotime trajectory.

Supplementary Fig. 6

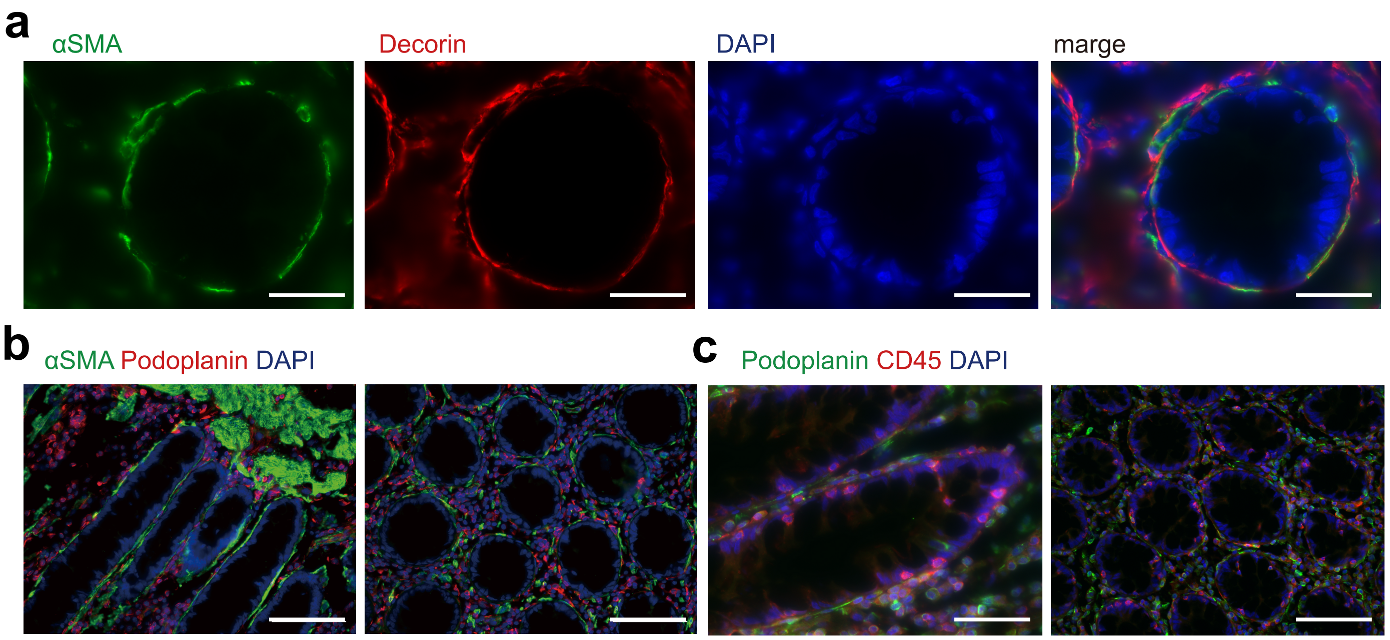

Supplementary Fig. 6: Immunofluorescence staining of myofibroblasts in human colon mucosa. **a.** Higher magnification of double labeling for fibroblast markers a-SMA (green) and Decorin (red) in the colonic mucosa. each fluorescent channel was displayed separately. Scale bar indicates 20 mm. **b.** Double labeling for a-SMA (green) and Podoplanin (red) in the colon mucosa. left: vertical section, scale bar indicates 100 mm; right: cross section, scale bar indicates 100 mm. **c.** Double labeling for Podoplanin (green) and CD45 (red). left: vertical section, scale bar indicates 50 mm; right: cross section, scale bar indicates 100 mm.

Supplementary Fig. 7

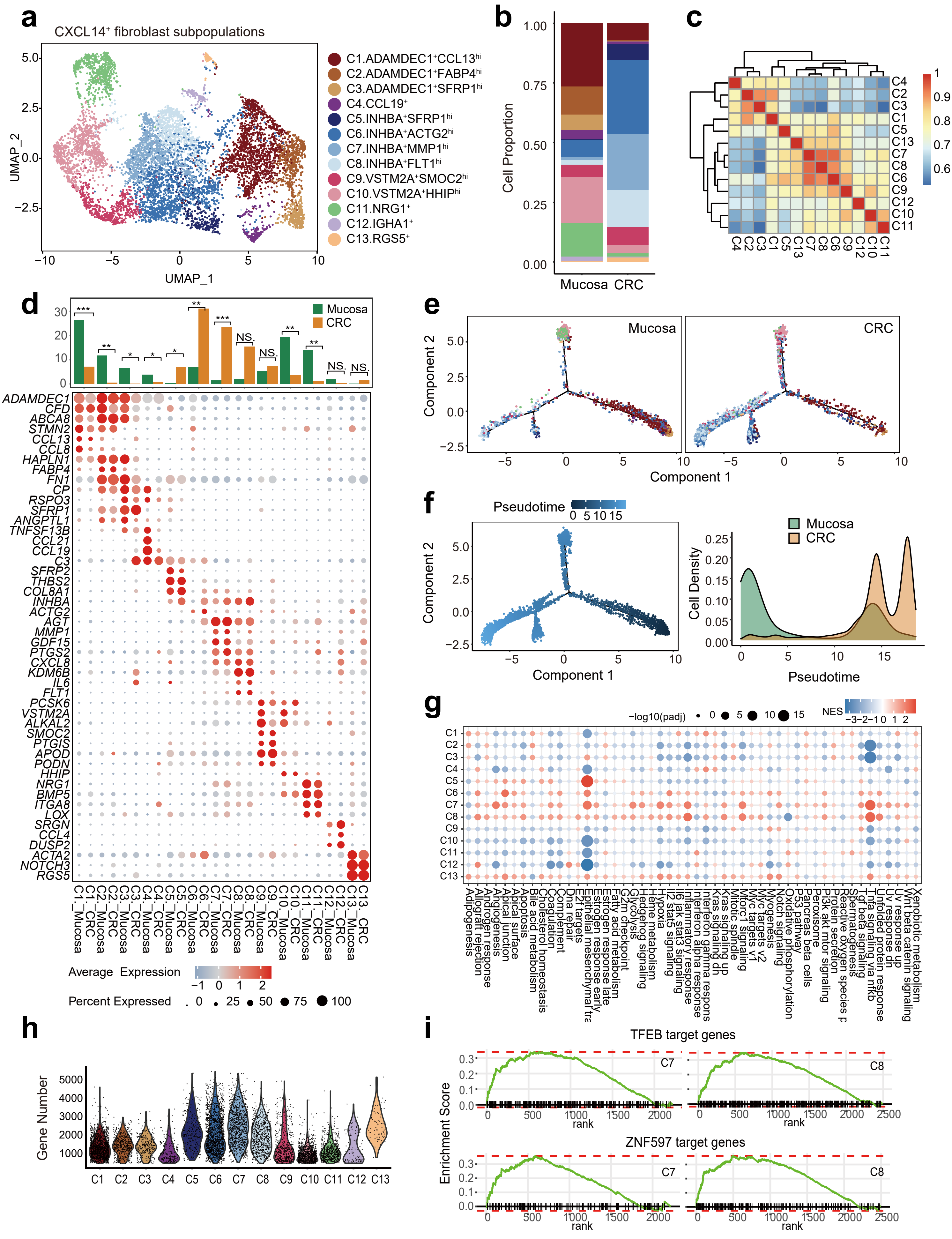

Supplementary Fig. 7: Subpopulations of CXCL14⁺ fibroblasts in human colon mucosa and in human colon cancer tissues.

a. UMAP projection of further analysis of $CXCL14^+$ fibroblasts. Cells were colored by new cluster identity. **b.** Comparison of $CXCL14^+$ fibroblasts composition between human colon mucosa and colon cancer tissues. **c.** Heatmap showing expression profile similarity between subclusters. **d.** Dot plot showing the scaled expression of signature genes for each $CXCL14^+$ subcluster in human colon mucosa and colon cancer tissues. The top histogram shows the proportional change in each subcluster. The p value was calculated by the t test. **e.** Trajectories of $CXCL14^+$ fibroblasts, displayed separately by sample source and colored by subcluster identity. **f.** left: Trajectories of $CXCL14^+$ fibroblasts color with pseudotime. right: Density of mucosal and colon cancer $CXCL14^+$ fibroblasts along the pseudotime trajectory. **g.** GSEA analysis of Hallmark Gene Sets of $CXCL14^+$ subclusters DEGs between CRC and mucosa. **h.** Violin plots showing the gene number of cells in each subcluster. **i.** GSEA plot showing Selected transcription factors which promote the formation of $INHBA^+$ subpopulations (C7, C8).
