# Supplementary material for: An integrated map of fibroblastic populations in human colon mucosa and cancer tissues
Source: Commun Biol. 2022 Dec 3;5:1326. doi: 10.1038/s42003-022-04298-5 (PMC9719516; doi:10.1038/s42003-022-04298-5)
Supplement: Supplementary file 3 — Description of Additional Supplementary Files [file 42003_2022_4298_MOESM3_ESM.pdf]

Description of Additional Supplementary Files

File name: Supplementary Data 1
Description: Cell counts and cell frequencies table for the fibroblastic populations in the normal human mucosa.

File name: Supplementary Data 2
Description: Table of marker genes for the human mucosa fibroblastic populations.

File name: Supplementary Data 3
Description: Table of marker genes for the fibroblastic populations in the integrated analysis of human mucosa and cancer tissue.

File name: Supplementary Data 4
Description: Cell counts and cell frequencies table for the fibroblastic populations in the integrated analysis of human mucosa and cancer tissue.

File name: Supplementary Data 5
Description: Differentially Expressed genes cancer tissue vs normal mucosa of each fibroblastic population.

File name: Supplementary Data 6
Description: Table of marker genes for the subpopulations of RGS5+ fibroblasts.

File name: Supplementary Data 7
Description: Cell counts and cell frequencies table for the subpopulations of RGS5+ fibroblasts.

File name: Supplementary Data 8
Description: Table of marker genes for the subpopulations of CXCL14+ fibroblasts.

File name: Supplementary Data 9
Description: Cell counts and cell frequencies table for the subpopulations of CXCL14+ fibroblasts.
