# Supplementary material for: An integrated map of fibroblastic populations in human colon mucosa and cancer tissues
Source: Commun Biol. 2022 Dec 3;5:1326. doi: 10.1038/s42003-022-04298-5 (PMC9719516; doi:10.1038/s42003-022-04298-5)
Supplement: Supplementary file 13 — Reporting Summary [file 42003_2022_4298_MOESM13_ESM.pdf]

nature portfolio

Corresponding author(s): Xianming Mo, Zongguang Zhou, Chuanwen Fan

Last updated by author(s): Nov 10, 2022

Reporting Summary

Nature Portfolio wishes to improve the reproducibility of the work that we publish. This form provides structure for consistency and transparency in reporting. For further information on Nature Portfolio policies, see our Editorial Policies and the Editorial Policy Checklist.

Statistics

For all statistical analyses, confirm that the following items are present in the figure legend, table legend, main text, or Methods section.

n/a	Confirmed	
☐	☒	The exact sample size (*n*) for each experimental group/condition, given as a discrete number and unit of measurement
☐	☒	A statement on whether measurements were taken from distinct samples or whether the same sample was measured repeatedly
☐	☒	The statistical test(s) used AND whether they are one- or two-sided<br>*Only common tests should be described solely by name; describe more complex techniques in the Methods section.*
☐	☒	A description of all covariates tested
☐	☒	A description of any assumptions or corrections, such as tests of normality and adjustment for multiple comparisons
☐	☒	A full description of the statistical parameters including central tendency (e.g. means) or other basic estimates (e.g. regression coefficient) AND variation (e.g. standard deviation) or associated estimates of uncertainty (e.g. confidence intervals)
☐	☒	For null hypothesis testing, the test statistic (e.g. *F*, *t*, *r*) with confidence intervals, effect sizes, degrees of freedom and *P* value noted<br>*Give P values as exact values whenever suitable.*
☒	☐	For Bayesian analysis, information on the choice of priors and Markov chain Monte Carlo settings
☐	☒	For hierarchical and complex designs, identification of the appropriate level for tests and full reporting of outcomes
☐	☒	Estimates of effect sizes (e.g. Cohen's *d*, Pearson's *r*), indicating how they were calculated

Our web collection on statistics for biologists contains articles on many of the points above.

Software and code

Policy information about availability of computer code

Data collection	BD Rhapsody WTA Local bioinformatics pipeline and Cell Ranger v3.1.0 (10X Genomics) was used to align and generate expression matrices for downstream analysis.
Data analysis	R(4.0.2), Seurat_4.1.0, ggplot2_3.3.6, monocle_2.22.0, clusterProfiler_4.2.0, DESeq2_1.34.0.

For manuscripts utilizing custom algorithms or software that are central to the research but not yet described in published literature, software must be made available to editors and reviewers. We strongly encourage code deposition in a community repository (e.g. GitHub). See the Nature Portfolio guidelines for submitting code & software for further information.

Data

Policy information about availability of data

All manuscripts must include a data availability statement. This statement should provide the following information, where applicable:

- Accession codes, unique identifiers, or web links for publicly available datasets
- A description of any restrictions on data availability
- For clinical datasets or third party data, please ensure that the statement adheres to our policy

The raw sequence data reported in this paper have been deposited in the Genome Sequence Archive (Genomics, Proteomics & Bioinformatics 2021) in National Genomics Data Center (Nucleic Acids Res 2021), China National Center for Bioinformation / Beijing Institute of Genomics, Chinese Academy of Sciences (GSA-

Human: PRJCA007492) that are publicly accessible at https://ngdc.cncb.ac.cn/gsa-human. All source data underlying the graphs and charts presented in the main figures have been presented in Supplementary Data.

Human research participants

Policy information about studies involving human research participants and Sex and Gender in Research.

Reporting on sex and gender	*Use the terms sex (biological attribute) and gender (shaped by social and cultural circumstances) carefully in order to avoid confusing both terms. Indicate if findings apply to only one sex or gender; describe whether sex and gender were considered in study design whether sex and/or gender was determined based on self-reporting or assigned and methods used. Provide in the source data disaggregated sex and gender data where this information has been collected, and consent has been obtained for sharing of individual-level data; provide overall numbers in this Reporting Summary. Please state if this information has not been collected. Report sex- and gender-based analyses where performed, justify reasons for lack of sex- and gender-based analysis.*
Population characteristics	*Describe the covariate-relevant population characteristics of the human research participants (e.g. age, genotypic information, past and current diagnosis and treatment categories). If you filled out the behavioural & social sciences study design questions and have nothing to add here, write "See above."*
Recruitment	*Describe how participants were recruited. Outline any potential self-selection bias or other biases that may be present and how these are likely to impact results.*
Ethics oversight	*Identify the organization(s) that approved the study protocol.*

Note that full information on the approval of the study protocol must also be provided in the manuscript.

Field-specific reporting

Please select the one below that is the best fit for your research. If you are not sure, read the appropriate sections before making your selection.

☒ Life sciences ☐ Behavioural & social sciences ☐ Ecological, evolutionary & environmental sciences

For a reference copy of the document with all sections, see nature.com/documents/nr-reporting-summary-flat.pdf

Life sciences study design

All studies must disclose on these points even when the disclosure is negative.

Sample size	We collected paired samples of human colon cancer tissues and the adjacent normal colon mucosa from 12 patients and dissociated each sample into single cells. The colon mucosa and colon cancer cell suspensions from 9 patients were sorted by fluorescence-activated cell sorting (FACS) to enrich CD326-, CD45- and CD31- cell populations and then generated single cell cDNA libraries with BD Rhapsody system for sequencing. The CD45- sorted colon mucosa cells and colon cancer cells from additional three pairs of specimens were generated single cell labelled cDNA libraries with Singleron GEXSCOPE for sequencing.
Data exclusions	We excluded the non-fibroblastic cell populations, including T cells, B/Plasma cells, epithelial cells, gut Gila cells, and dendritic cells, endothelial cells, , and a small fraction of mast cells.
Replication	The key findings of the paper were confirmed by multiple complementary experiments.
Randomization	No randomization of subjects was performed in this study.
Blinding	The integration of patients colon cancer tissues and the adjacent normal colon mucosa were performed without bias about expected results.

Reporting for specific materials, systems and methods

We require information from authors about some types of materials, experimental systems and methods used in many studies. Here, indicate whether each material, system or method listed is relevant to your study. If you are not sure if a list item applies to your research, read the appropriate section before selecting a response.

Materials & experimental systems

n/a	Involved in the study
☐	☒ Antibodies
☒	☐ Eukaryotic cell lines
☒	☐ Palaeontology and archaeology
☒	☐ Animals and other organisms
☒	☐ Clinical data
☒	☐ Dual use research of concern

Methods

n/a	Involved in the study
☒	☐ ChIP-seq
☐	☒ Flow cytometry
☒	☐ MRI-based neuroimaging

Antibodies

Antibodies used

anti-RGS5 (Proteintech, 11590-1-AP), anti-decorin (Proteintech, 14667-1-AP), anti-podoplanin/gp36 (Abcam, ab10288), anti-CD36 (Beyotime, AF6414), anti-actin (C-2) (SANTA, sc-8432), anti-Hhip (5D11) (SANTA, sc-293265)), anti-CD326-PE-Cyanine7(25-9326-42), anti-CD45-BV510 (Cat 563204), and anti-CD31 (PECAM-1)(89C2) (Cell Signaling, #3528),anti-CD45 (proteintech, 80297-1-RR)

Validation

Antibodies were validated by the manufacturers.
anti-RGS5 : https://www.ptglab.com/products/RGS5-Antibody-11590-1-AP.htm
anti-decorin: https://www.ptglab.com/products/DCN-Antibody-14667-1-AP.htm
anti-podoplanin/gp36: https://www.abcam.com/podoplanin--gp36-antibody-18h5-bsa-and-azide-free-ab10288.html
anti-CD36 (Beyotime, AF6414): https://www.beyotime.com/mobilegoods.do?method=code&code=AF6414
anti-actin (C-2) : https://www.scbt.com/p/actin-antibody-c-2
anti-Hhip (5D11) :https://www.scbt.com/p/hhip-antibody-5d11
anti-CD326-PE-Cyanine7: https://www.thermofisher.cn/cn/zh/antibody/product/CD326-EpCAM-Antibody-clone-1B7-Monoclonal/25-9326-42
anti-CD45-BV510: https://www.bdbiosciences.com/ja-jp/products/reagents/flow-cytometry-reagents/research-reagents/single-color-antibodies-ruo/bv510-mouse-anti-human-cd45.563204
anti-CD31: https://www.cellsignal.com/products/antibody-conjugates/cd31-pecam-1-89c2-mouse-mab-alexa-fluor-555-conjugate/61255
Anti-Cytokeratin 20:https://www.abcam.com/cytokeratin-20-antibody-cytoskeleton-marker-ab97511.html
anti-CDX2:https://www.labome.com/product/Dako/M3636.html
anti-CD45: https://www.ptglab.com/products/CD45-Antibody-80297-1-RR.htm

Flow Cytometry

Plots

Confirm that:

☒ The axis labels state the marker and fluorochrome used (e.g. CD4-FITC).

☒ The axis scales are clearly visible. Include numbers along axes only for bottom left plot of group (a 'group' is an analysis of identical markers).

☒ All plots are contour plots with outliers or pseudocolor plots.

☐ A numerical value for number of cells or percentage (with statistics) is provided.

Methodology

Sample preparation

To obtain single-cell suspensions, samples were cut into small pieces (approximately 1 mm3) and digested with a defined cell dissociation solution containing collagenase Ⅰ (A004194), collagenase Ⅱ (A004174), collagenase Ⅳ (A004186), dispase (A002100) and DNase I (CAS 9003-98-9) for 40-60 min at 37 °C. Cells were then filtered through a 40-μm cell strainer and resuspended at a final concentration of approximately 5×106 cells/ml.

Instrument

BD FACSAria II

Software

FlowJo (v.10.8)

Cell population abundance

Fluorescent microscopic detection,over 95%

Gating strategy

To distinguish negative and positive events, unstained samples were used as negative control for each antibody. The sample containing only buffer without cells was used as negative control for FSC/SSC gates of starting cells.

☒ Tick this box to confirm that a figure exemplifying the gating strategy is provided in the Supplementary Information.
